# Developments in toxicogenomics: understanding and predicting compound-induced toxicity from gene expression data

**DOI:** 10.1039/c8mo00042e

**Published:** 2018-06-19

**Authors:** Benjamin Alexander-Dann, Lavinia Lorena Pruteanu, Erin Oerton, Nitin Sharma, Ioana Berindan-Neagoe, Dezső Módos, Andreas Bender

**Affiliations:** a University of Cambridge , Centre for Molecular Informatics , Department of Chemistry , Lensfield Road , Cambridge CB2 1EW , UK . Email: dm729@cam.ac.uk ; Email: ab454@cam.ac.uk; b Babeş-Bolyai University , Institute for Doctoral Studies , 1 Kogălniceanu Street , Cluj-Napoca 400084 , Romania; c University of Medicine and Pharmacy “Iuliu Haţieganu” , MedFuture Research Centre for Advanced Medicine , 23 Marinescu Street/4-6 Pasteur Street , Cluj-Napoca 400337 , Romania; d University of Medicine and Pharmacy “Iuliu Haţieganu” , Research Center for Functional Genomics , Biomedicine and Translational Medicine , 23 Marinescu Street , Cluj-Napoca 400337 , Romania; e The Oncology Institute “Prof. Dr Ion Chiricuţă” , Department of Functional Genomics and Experimental Pathology , 34-36 Republicii Street , Cluj-Napoca 400015 , Romania

## Abstract

The toxicogenomics field aims to understand and predict toxicity by using ‘omics’ data in order to study systems-level responses to compound treatments. In recent years there has been a rapid increase in publicly available toxicological and ‘omics’ data, particularly gene expression data, and a corresponding development of methods for its analysis. In this review, we summarize recent progress relating to the analysis of RNA-Seq and microarray data, review relevant databases, and highlight recent applications of toxicogenomics data for understanding and predicting compound toxicity. These include the analysis of differentially expressed genes and their enrichment, signature matching, methods based on interaction networks, and the analysis of co-expression networks. In the future, these state-of-the-art methods will likely be combined with new technologies, such as whole human body models, to produce a comprehensive systems-level understanding of toxicity that reduces the necessity of *in vivo* toxicity assessment in animal models.

## Abstract 2

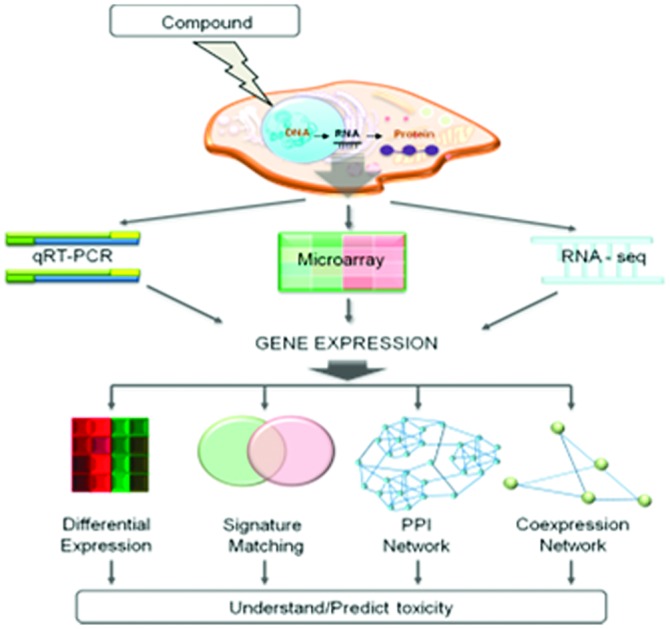
This review highlights developing methods in the toxicogenomics field and their applications to understanding and predicting compound induced toxicity.

## Introduction

Compound toxicity is one of the major contributors to the high clinical attrition rates of new drug candidates, with lack of safety being the cause of 24% of failures between 2013–2015.^1^,^2^ Anticipating the toxicity profile of a new chemical entity in humans is not an easy task, as it is hampered by long experimental durations and associated high costs of long-term toxicity studies, as well as our reliance on the use of animal studies to measure adverse effects, which are often not sufficiently predictive for predicting toxicity in humans.^3^,^4^ In recent years, there has been intense effort to improve upon the current situation, as developments in predicting and understanding toxicity would reduce the need for animal testing and improve the attrition rate in drug development, which is an essential goal for the pharmaceutical industry in the near future.^5^

One approach to address this has been to treat toxicity as a ‘Systems Biology’ problem, considering activity in the whole system simultaneously, as opposed to *e.g.* activity against a single receptor. Whilst the concept of systems biology is over 50 years old,^6^ only recently have advancements in high-throughput technologies led to the generation of sufficiently large data sets to assess the state of a biological system in a meaningful way.^7^ These data types (and the techniques used to analyse them) are generally grouped under the ‘omics’ label. The current major ‘omics’ techniques are genomics, transcriptomics, metabolomics, and proteomics.^8^,^9^ In an ideal world, toxicogenomics would integrate these four types of readouts, in addition to other (future) omics layers of biological information, thereby capturing a closer approximation to the ‘complete’ biological response of a system to a compound treatment.^10^ The direct biological measurement of compound activity in different cell lines and organs offered by these various omics techniques is complementary to the structure-based viewpoint of compounds in drug development, and thus can be a valuable tool in assessing potential toxicity.^11^ However, the integration of multiple omics in toxicity prediction has only been achieved in a handful of studies.^12^–^14^

As such, the definition of toxicogenomics varies: the American National Research Council defines toxicogenomics as “combin[ing] toxicology with information-dense genomic technologies to integrate toxicant-specific alterations in gene, protein and metabolite expression patterns with phenotypic responses of cells, tissues and organisms”.^15^ On the other hand, Creasy and Chapin limit toxicogenomics to only “the study of altered gene expression after toxicant exposure”.^16^ Whilst this narrows the definition, gene expression provides a detailed snapshot of the response of the biological system to a compound treatment and, with relatively mature experimental technology as well as established methods of data analysis, it possesses (in the opinion of the authors) a practically useful (albeit variable) cost/signal ratio. Further, large amounts of gene expression data are now available in the public domain, enabling new biological questions to be addressed through data re-use, without the need for further experimentation. Hence, in this review, we will specifically discuss the utilization of transcriptomics data in the toxicogenomics field.

Progress in this field has previously been hampered by a lack of large-scale, suitable, public databases. This changed in 2011 when both DrugMatrix^17^ and Open TG-GATEs^18^ were made public. Both databases interweave compound-induced gene expression data with *in vivo* histopathological data (see later for full description). These toxicogenomics databases are complemented by other large-scale transcriptomics databases, such as the Connectivity Map^19^ and the Library of Integrated Network-based Cellular Signatures L1000 dataset (LINCS),^20^ that link compounds to gene expression responses in cell lines. Additionally, the Comparative Toxicogenomics Database provides compound-gene-phenotype associations.^21^

This available data enables the elucidation of the mode of action of a compound treatment, as well as the identification of toxicity-related biomarkers. However, this is limited by the strength of the transcriptomic signal (assuming that a meaningful transcriptomic response exists), and our ability to discover a signal in such noisy, high-dimensional data. Toxicity related biomarkers and efficacy related transcriptomics signals are important for clinical candidate selection as they aid compound evaluation at an early stage in drug development.^22^

The field of toxicity itself can be split into many areas, with those that mainly concern compound treatments generally falling into the classes of genotoxicity and organ toxicity.^23^ This review will mainly focus on the latter, without reference to a specific definition of toxicity, which naturally differs from study to study.

**Fig. 1:**
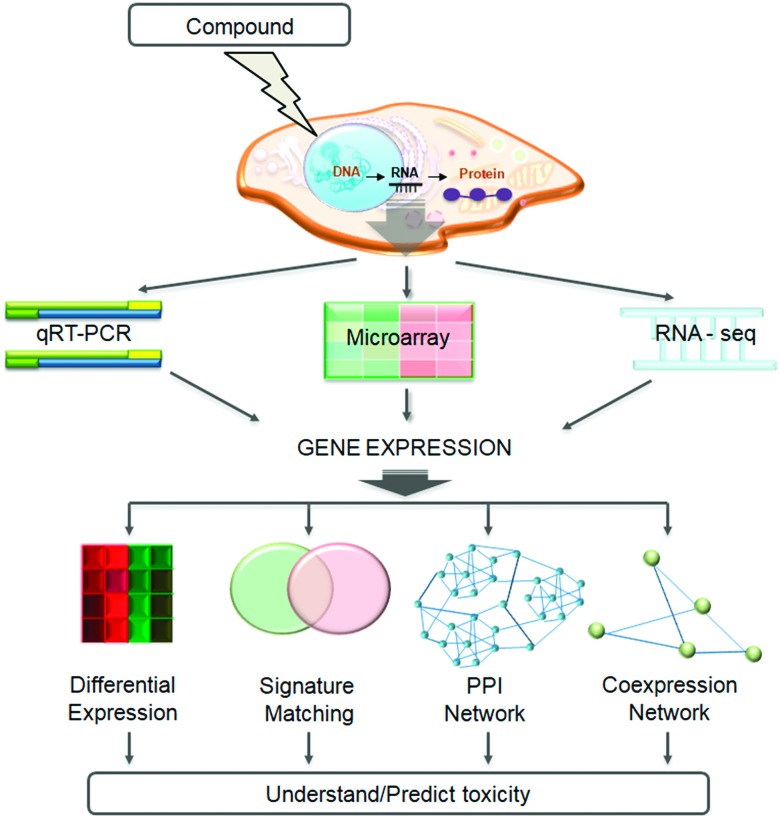
Methods and technologies utilized in the toxicogenomics field. The figure represents the use of qRT-PCR, microarray, and RNA-Seq methods to measure transcriptomic response, which in the context of this review may refer to the response to compound treatment, or the comparison of diseased/toxic and healthy states. Measured gene expression can then be analysed, using various computational methods, to understand and predict toxicity. These methods include differential gene expression analysis, gene expression signature matching, protein–protein interaction network (PPI network), and co-expression network analysis.

In this review, we shall cover the generation of transcriptomic data and summarize the available databases related to toxicogenomics studies. We go on to describe the state-of-the-art methodologies developed to utilise these data for understanding and/or predicting toxicity, and discuss case studies from the field. We will focus on four main methods (shown in Fig. 1): differential gene expression analysis, compound signature matching, utilising protein–protein interaction networks, and creating and analysing gene co-expression networks.

## Experimental approaches to measure gene expression

A number of different methodologies have been utilized, individually or in combination, to determine the transcriptomic profile changes of a biological system after a perturbation.^8^ The most commonly employed techniques are real-time quantitative polymerase chain reaction (RT-qPCR), microarray analysis and, more recently, RNA sequencing (RNA-Seq, Fig. 1). These methods all have advantages and disadvantages, as described in detail by Bourdon-Lacombe *et al.*^3^ RT-qPCR is the most sensitive out of the three but also the most time-consuming. It can be used only for a limited number of genes, so it is used mostly for validation. Hence, in order to understand the toxic effects of a compound at a systems level, microarray, and more recently RNA-Seq, are the preferred technologies which will be described in more detail in the following section.

DNA microarrays were first developed and employed in the 1990s.^24^ In short, microarrays use nucleotide sequences bound to a chip, called probes. To these probes bind the fluorescently tagged reverse transcribed sample cDNA. The location and strength of the induced fluorescence indicate which RNA is detected and in what abundance.^25^ This makes the resultant intensity value continuous. As the probes are designed with specific nucleotide sequences, microarrays are not able to detect unknown transcripts, which renders the technique often unsuitable for lesser known areas of transcriptome space such as lncRNA or all miRNA detection. Also, the reverse transcribed cDNA can bind to probes other than its exact matching probes (cross-hybridization) which may result in a higher observed expression value compared to the real expression of the gene, potentially leading to inaccuracies of measurement.^26^ Nevertheless, different microarray platforms and laboratories can detect concordant biological signals,^27^,^28^ illustrating the ability of microarrays to capture relevant transcriptomic responses. A further advantage of microarray technology is that it is a relatively mature technology, with numerous well-established commercial and open source data analysis tools.^29^,^30^ Microarrays have been widely applied in toxicogenomic studies,^31^,^32^ including the use of measured gene expression to build machine learning models which produce coherent results predicting toxicity.^33^

Rather than detection of fluorescence, RNA-Seq is based on counting reverse transcribed cDNA. RNA-Seq techniques are capable of detecting *de novo* sequences and different RNAs from one sample (*e.g.* mRNA, miRNA, lncRNA, snRNA *etc.*).^34^ A typical differential expression pipeline starts with performing a quality check (QC) of the cDNA reads. The reads passing the QC step are mapped to a reference genome or transcriptome. This is followed by quantification, to measure how much of each gene is transcribed under particular conditions.^35^,^36^ Many tools/R packages are available for each stage of an RNA-Seq workflow, leading to multiple potential analysis pipelines. Whilst some general guidelines (resulting either from analytical or practical considerations) do exist,^37^–^42^ there is no definitive consensus with respect to *e.g.* statistical methods to be used in a given context. An advantage of RNA-Seq is its lower detection limit compared to microarrays;^43^ a further difference between the two methodologies is that microarrays measure continuous values whereas RNA-Seq read counts are discrete,^35^ necessitating novel statistical methods in RNA-Seq data analysis. RNA-Seq technologies are currently more expensive, but the gap is closing. A complete RNA-Seq experiment is between a few hundred and a few thousand dollars meanwhile microarrays are around a few hundred dollars per sample.^44^

The use of different statistical analysis methods and normalization processes has a non-negligible effect on the measured expression values,^45^ so careful consideration of these factors is advisable. For reasons of scope, we are only able to provide a brief overview of experimental and pre-processing techniques here; we refer the reader to several detailed reviews and advice on how to design experiments and analyse microarray and RNA-Seq data which have been published previously.^30^,^46^,^47^

## Toxicogenomic databases

Progress in toxicity prediction will always depend on the amount and quality of available data. There are three main public databases in the field that directly associate toxicity and gene expression data: DrugMatrix,^17^ Open TG-GATEs (Toxicogenomics Project-Genomics Assisted Toxicity Evaluation System)^18^ and the Comparative Toxicology Database (CTD),^48^ which are listed (along with other related databases) in Table 1.

**Table 1:** Different repositories of compounds induced transcriptomics response databases

Database	Cell lines/tissues	Number of unique compounds (unique signatures)	Time points/doses	Platform	Metadata	Publication year	Reference/website
DrugMatrix^50^	*In vivo* rat data; liver, kidney, heart and thigh muscle	627 (5288)	Repeat dose and single dose studies; 6 h, 24 h, 3 day, 5 day	Affymetrix GeneChip Rat Genome 230 2.0 Array GE Codelink™ 10 000 gene rat array	Histopathology, blood chemistry, clinical chemistry	2006 (originally)	ftp://anonftp.niehs.nih.gov/drugmatrix/
						2011 (publicly)	
Open TG-GATEs^18^	*In vivo* rat; kidney and liver	170 (2400)	Repeat dose and single dose studies	Affymetrix GeneChip Rat Genome 230 2.0 Array	Histopathology, blood chemistry, clinical chemistry	2015	http://toxico.nibio.go.jp/english/index.html
	*In vitro* human and rat primary hepatocytes						
Connectivity map^19^	5 human cancer cell lines	1309 (6100)	Predominantly single dose, 6 hour time point	Affymetrix GeneChip Human Genome U133A Array	None	2006	https://clue.io/broadinstitute.org/cmap
				22 283 probe sets			
Library of Integrated Network-based Signatures L1000 dataset (LINCS)^51^,^52^	Up to 77 cell lines	>27 927 compound signatures	Various, mainly 6, 24, 96 and 144 hours	Proprietary Broad L1000 assay measures 978 ‘landmark transcripts’ and 80 invariant ‘control transcripts’	Microscopy images	2014 (phase 1)	https://clue.io/
						2017 (phase 2)	

DrugMatrix was originally produced as a commercial database in 2006 and transferred into the public domain in 2011. It contains gene expression response to compound treatments in rat tissues. The structure of the database is summarized in Table 1 and has been described in detail in previous work.^17^ DrugMatrix is a valuable resource as it contains compound induced gene expression over a number of tissues. Crucially, it also provides histopathological, hematologic and clinical chemistry data associated with compound treatments, allowing specific forms of toxicity to be investigated. Additionally, it anchors gene expression changes to the resultant phenotype.^49^

Open TG-GATEs^18^ was created following a similar protocol to DrugMatrix and also contains both gene expression data and histopathology data from different rat tissues. It focuses on time course studies using repeated doses, which allows the chronic effect of toxicants to be followed. It should be noted that, while most of the experimental setups are the same, the doses used in DrugMatrix and Open TG-GATEs are not. The maximum tolerated dose in DrugMatrix is defined as that which causes a ‘50% reduction in weight gain over control after 5 days of daily dosing’^50^ and in general, two doses were used in the generation of DrugMatrix data. On the other hand, TG-GATEs defines its highest dose as that which induces ‘the minimum toxic effect over the course of a 4 week toxicity study’; three doses were then used in both the repeat and single-dose studies, with each study performed in triplicate.^18^ This difference in dosing reflects the compound selection and experimental setup of the two databases: TG-GATEs includes compounds that had previously been annotated in the literature with a toxic effect, whereas DrugMatrix aimed to cover a more diverse chemical space. As such, DrugMatrix might often require a higher dose to see a toxic phenotype.

The CTD consists of pairwise interaction data between chemicals, genes, and diseases that have been manually curated and inferred from literature.^48^ The curated data is collected from over 564 species and each species is shown when querying the database. There are also smaller databases for more specific toxicities such as for drug-induced liver injury.^53^

In addition to the above databases, which connect toxicity readouts with the gene expression response *in vivo* animal tissues, there are several databases which contain compound-induced gene expression responses. These include Connectivity Map (CMap),^19^ the Library of Integrated Network-based Cellular Signatures L1000 dataset (henceforth referred to as LINCS),^51^,^52^ ArrayExpress and the Gene Expression Omnibus (GEO) (Table 1).^54^,^55^

The CMap project started in 2006 with gene expression profiles of 164 small-molecule compounds and was later updated to build 2, containing expression profiles of 1309 drugs across five cell lines (see Table 1).^19^ LINCS was created as a large-scale expansion of the original CMap and, at the time of writing, the LINCS project has reached its second phase, in which nearly 20 000 small-molecules, as well as other perturbagens including shRNAs, cDNAs and biologics, have been profiled on up to 77 cell lines.^20^,^52^ Expression profiling on this scale was made possible by the use of the L1000 platform, which aims to capture the greatest amount of variation while measuring only a subset of 978 genes.^52^ This subset of genes was chosen to capture the greatest proportion of the variance in expression, allowing (in principle) the prediction of the expression of at least 80% of non-measured transcripts, the accuracy of which however depends on data quality.^52^,^56^,^57^

GEO and ArrayExpress are general purpose (non-toxicity specific) repositories that contain user uploaded data, and so are continually updated. Both databases contain a wide range of experiments covering compound treatments, diseases, and other conditions, across different platforms and species. ArrayExpress^54^ is checked by curators meanwhile GEO^58^ is user uploaded, so the former has higher quality standards, and fewer uploaded studies (70 878 studies *vs.* 96 622 at 4th of April, from 614 in ArrayExpress and 975 in GEO with the keyword “toxicity”). The gene expression data from both DrugMatrix and Open TG-GATES are available from ArrayExpress; the data from LINCS is also available from GEO.

The available *in vivo* data is necessarily limited to mouse and rat models, whereas various human cell lines have been used in other studies. When analysing and interpreting data from these repositories, the difference between specific cell lines and animal models should be taken into consideration, as it will play a significant role in the biological meaning of the toxic response. Despite these considerations, the availability and size of these public databases allow for the development of methods that enable the identification of the biological processes taking place *in vivo* and *in vitro*. These methods will be now investigated in the following sections.

## Current systems biology methods used in toxicogenomics

### Differential gene expression analysis

**Fig. 2:**
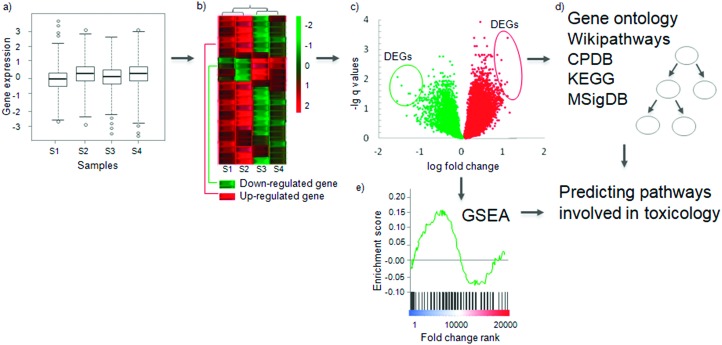
Determining differentially expressed genes and conducting pathway analysis. (a) The log-transformed gene expression distribution of normalized samples. (b) Differences in the expression profiles of a gene across samples between two experimental conditions, *e.g.* toxicant-exposed and not-exposed, on a heat map. Each row indicates one gene and a sample is indicated by a column (samples S1, S2, S3, S4). Green indicates lower expression (down-regulated) and red indicates higher expression (up-regulated). Samples are clustered according to the expressed genes by hierarchical clustering. Using such clustering can show cell line- or tissue-specific responses to compounds. (c) Representative vulcano plot as a result of gene expression analysis. The circled genes represent those genes which meet selected threshold of statistical significance (*q* < 0.1) and fold change (abs log 2 FC > 1) – Differentially Expressed Genes (DEGs). (d) The DEGs can be searched for enrichment in pathways from different databases including Gene Ontology, CPDB (Consensus Pathway Database), Wikipathways, KEGG (Kyoto Encyclopedia for Genes and Genomes) or MSigDB (Molecular Signature Database). See details in the subsequent section and Table 3. (e) Another method of analysis, Gene Set Enrichment Analysis (GSEA) uses the whole profile of genes (rather than just the DEGs) to discover pathway enrichment. The final output of both methods will be pathways which are involved in that particular toxicological response to a compound treatment.

Once gene expression values have been determined experimentally (Fig. 2a), for a sample and a control condition, the next step in a gene expression analysis aims to determine the Differentially Expressed Genes (DEGs). A gene is considered to be differentially expressed if the observed difference between two experimental conditions is statistically significant.^59^ The exact definition of significant differential expression depends on the underlying mathematical model and assumptions used, which are summarized in Table 2. The methods can be broadly categorised into two types: those that consider a single gene's expression values, such as fold change and rank product methods, and those that utilise the gene expression values’ entire distribution, such as Bayesian and counting methods. Most of the methods are compatible with both RNA-Seq and microarrays, but those which require exact counts are not suitable for microarrays.

**Table 2:** Methods to determine differentially expressed genes

Method	Description	Comment	Example packages using the method
Fold change	– Calculates the ratio of a gene's expression between sample and control	– Works with small sample size	limma^64^,^65^
	– Genes are classed as differentially expressed according to a selected threshold (usually an absolute log-fold change value greater than 0.5 to 2)	– Easy to interpret	WAD^66^
	– Usually used in conjunction with a non-parametric/linear/Bayesian significant test	– Does not take into account the sample variance	
		– Different ways to calculate depending on the use of averages, medians, *etc.*	

Non-parametric tests	– Rank-product method, Mann whitney *U* tests for comparing two categories	– Capable to compare different platforms’ results	RankProd^68^,^69^
	– Kruskal–Wallis test for multiple categories	– RankProd is best method for meta analysis^67^	
	– Compares the ranks of the genes according to their expression		

Linear methods (*t*-test, ANOVA)	– Compare the mean value of expression per gene in samples	– Add statistical significance, but uses the boundary condition the gene expression values of conditions are normally distributed	Cuffdiff 2^70^
	– The null hypothesis is that the means are equal – *t*-test is for two category comparison	– Commonly used with fold change	limma – after a Bayes procedure
	– ANOVA is for multiple categories		

Bayesian methods	– Use the data to predict the probabilities of differential expression	– Have relatively high computation time dependence	limma
	– Use the standard deviation to alter the test statistics or tests directly	– Makes more appropriate results then a *t*-test	baySeq^71^

Counting method	– Uses the real count of the expressions for comparison with a negative binomial test	– Requires exact number of mRNA copies	DESeq2^65^
			edgeR^72^

The most common approach is the fold change method (Fig. 2b), which calculates the differential expression between sample and control. Then to obtain statistical significance a false discovery rate corrected *t*-test is used.^60^ In early works and especially in the case of small sample size DEG determination relied only on fold change (FC). This methodology lacks statistical tests for differentially expressed genes so using only FC has to be avoided in any gene expression experiment and a minimum sample size of three per condition should be used to capture the biological variance. Selected cut-offs may then be applied for both significance and fold change. Thresholds vary from study to study, but common threshold choices include *q* values (*i.e.*, multiple-testing corrected significance values) <0.1, and absolute log 2 based fold change > 1. The patterns of expression change across different sample groups can be visualised using a heatmap (Fig. 2b); another visualization method is the volcano plot, which also shows the statistical significance of the fold changes (Fig. 2c). The DEGs can be used as markers for a mode of toxicity, or as variables in a predictive model to predict whether a compound is toxic or not.^61^–^63^

In the following, we will outline some examples of the use of differential expression in the toxicogenomics field. In order to identify DEGs following administration of a known toxicant, MPP+ (1-methyl-4-phenyl-pyridinium), in human neuroblastoma cells, microarray analysis was performed by Conn *et al.*^73^ They defined DEGs as genes which have higher than 1 FC and confirmed them by RT-qPCR. Among those, two transcription factors, namely c-Myc proto-oncogene and RNA-binding protein 3, were found to be associated with MPP+ toxicity. The mode of toxicity of MPP+ exposure was investigated further in a time-dependent manner utilizing the EDGE (Extraction of Differential Gene Expression) program.^74^,^75^ 79 DEGs were found passing the strong cut off *q*-value threshold of 0.001. Different histones such as H2AFJ, H3F3B, HIST1H2AC, HIST1H2BD, HIST1H2BG, HIST1H2BK, showed differential expression, suggesting that toxicity seems to be related to the destabilization of nucleosomes after the initial exposure to MPP+ in the neuroblastoma cells.

A popular choice for DEG analysis in microarray^76^–^78^ and RNA-Seq^43^,^60^ data is the limma package, which provides rich features with linear modelling and Bayesian estimates of which also consider the variance of the genes per sample. This helps the statistical prediction to have more power. As an example, crystalline silica was studied in regard to pulmonary toxicity effects on human A549 lung adenocarcinoma cells *in vitro* and *in vivo* rat lungs. Here, microarray data were analyzed with the limma package, considering fold change and a Bayesian statistics predicted *t*-test value in order to identify DEGs. The authors found concordance in the affected pathways between rat lungs and human A549 cell lines (see next section, Fig. 2d).^77^ Significantly overexpressed genes suggested potential novel mechanisms in pulmonary toxicity induced by silica. These genes were *e.g.* different dual specific phosphatases (DUSP1 and 5) or the growth arrest proteins GADD34, GADD45α. The same approach was used to identify DEGs for melphalan-induced vascular toxicities in a human retinal endothelial cell model.^78^ The authors constructed a transcription factor target network (see network section) to analyse gene signatures and predicted five potential drug candidates that could potentially avoid this type of toxicity by targeting transcription factors, such as MYC and JUN, directly. This study illustrates how the understanding of compound toxicity can also suggest novel hypotheses of efficacious medicines, although prospective validation was not performed in this study.

Rank product methods, which are platform independent and non-parametric have also been successfully used in the study of toxicity.^67^,^79^,^80^ In these methods, genes are ranked according to their expression and compared between case and control based on their rank, rather than the magnitude of fold-change or *t*-test significance values. In the case of tubule toxicity, work by Shi *et al.* compared the rank product method with three other differential expression measuring algorithms (*t*-statistics, fold-change, and *B*-statistics) and their combinations to predict rat nephrotoxicity using the 20 most differentially expressed genes.^80^ They found rank product methods models gave the most specific (96.7%) and accurate (89.7%) results, however, it was not as sensitive (66.7%). In contrast, the combination of *t*-test and fold-change gave the most balanced performance in the sense of specificity, accuracy, and sensitivity (83.6%, 81.0%, 72.2% respectively). DEGs, including PPAR, RXR, and D vitamin receptor, were found to be involved in tubule toxicity pathways.

The rank product method was also used in a meta-analysis study by Yim *et al.* with the aim to find novel biomarkers of volatile organic compound toxicity in human hepatocellular carcinoma cells.^81^ The significantly overexpressed genes were ribosomal proteins RPL27, RPS6, RPS11, RPS27A, heat shock protein 60, a farnesyltransferase and aurora kinase, genes that showed to be related to various respiratory symptoms.

More recent toxicogenomic studies often use RNA-Seq data, which provide quantitative information and hence in many cases better resolution than microarrays. The simple *t*-test using Cufflinks^82^ (Table 2) was used to investigate the effect of fluoride exposure on the testicles of healthy male mice. This resulted in 367 DEGs and shed light on the involvement of IL17 in fluoride's mechanism of toxicity, and hence improved understanding of this effect.^83^

RNA-Seq methods allow comparison of the exact count of the transcripts, which is used by the edgeR^72^ and DESeq^84^ packages. edgeR was used to determine DEGs in human airway epithelial cells exposed to the *Streptococcus pneumoniae* toxin pneumolysin and the preventive effect of statins.^85^ They showed the differentially expressed genes form a network around 4 transcription factors: sterol regulatory element-binding transcription factor 1 and 2 and early growth response gene 1 and 2. They conducted KEGG pathway and Gene Ontology Biological Process enrichment analysis (see the next section) which emphasized the role of lipid metabolism in pneumolysin exposure and the protective effects of statins.

A further study tested the effect of aflatoxin B1 on *in vivo* male rat liver, comparing the results of DESeq and Cuffdiff analysis using RNA-Seq data to results of *t*-test using microarray data.^45^ DESeq analysis resulted in 1026 differentially expressed transcripts meanwhile Cuffdiff showed only 119 and *t*-tests on microarrays 626 such transcripts. The results of DESeq included 49 novel transcripts which were confirmed by qPCR.^45^ Additionally, Kovalova *et al.* tested the effect of 2,3,7,8-tetrachlorodibenzo-*p*-dioxin on three species (mouse and rat *in vivo* and human B cells *in vitro*) using RNA-Seq and DESeq algorithm. The cytochrome P450 isoenzyme CYP1A1 had concordant increased expression regardless of the species and tissue.^86^

Although differentially expressed genes often represent a good start for determining the biological reasons for the toxic effects of a compound, a direct analysis of gene expression space often suffers from high dimensionality and noise of the individual gene measurements. It should be noted that some compounds do not strongly affect gene expression, resulting in a transcriptional signal which is dominated by noise rather than reflecting the effect of the compound on biological processes.^87^,^88^ Hence, using additional analysis, such as biological pathway enrichment, can aid distinguishing signal from noise. These methods are described in the next section.

### Pathway analysis

Once the differentially expressed genes have been determined, the most common analytical method is pathway analysis to figure out which ‘biological functions’ are altered after compound exposure (Fig. 2d and e). However, the definition of ‘pathways’ or ‘biological functions’ depends on the database used. These definitions are evolving and may even be considered as somewhat arbitrary.^89^ Pathways are species dependent and so care must be taken when using the databases to ensure that the appropriate organism-specific pathway or ontology databases are available. The two most common analytical methods to determine the differentially expressed pathways or functions are the simple hypergeometric enrichment test, and Gene Set Enrichment Analysis (GSEA, Fig. 2d and e).^90^ The difference between the two lies in the null hypothesis. The hypergeometric enrichment test (reviewed recently^91^) investigates whether a pathway or a biological function occurs more often in DEGs compared to an appropriate background: usually either the set of genes measured in the microarray/RNA-Seq experiment or the entire genome of the species in the database (Fig. 2d). The null hypothesis is that the genes of a pathway are not enriched in the DEGs. Therefore, this method requires a predefined cut-off for determining which genes are significantly differentially expressed (see previously). GSEA, on the other hand, uses the expression value of all measured genes. It ranks the genes according to a metric (*e.g.* fold change) and then determines whether the genes from a set (*e.g.* from a pathway) occur in the high or low end of the ranked list. The null hypothesis here is that the genes from the set occur randomly in the ranked list. GSEA uses a Kolmogorov–Smirnov test for statistical significance of the enrichment. GSEA does not require a pre-defined cut-off to be specified for DEGs, in contrast to simple enrichment analysis (Fig. 2e).^90^

Both methods require gene sets for testing. Such gene sets can be obtained from the different pathway databases available, some of which are summarized in Table 3. Many of the toxicogenomic studies mentioned earlier have used pathway analysis, illustrating how this can be carried out using different pathway databases after selection of DEGs. Gene Ontology^74^,^99^ (GO) is probably the most commonly used database.^100^–^102^ However its hierarchical nature, as well as the nonspecificity of the higher GO layers, lead to difficulty in interpretation. To avoid such difficulties, it is good practice according to the authors' experience to group the annotations and identify the common grounds of the found GO terms; this feature is found in many online GO enrichment tools.

**Table 3:** Pathway databases for toxicogenomic studies

Database	Description	Comment	Link
WikiPathways^92^	Integrated collection of different pathway databases	Freely available, everyone can curate	https://www.wikipathways.org/
Reactome^93^	Large database with a focus on signaling pathways	Free and the largest database of its kind	https://reactome.org/
Gene Ontology^94^	Gene product functional annotation in a hierarchically structured ontology	Contains annotations at multiple levels of specificity	http://www.geneontology.org/
Reviewed:^95^			
Kyoto Encyclopedia of Genes and Genomes^96^	One of the oldest pathway databases; content constantly updated	Very good metabolic pathway collection, but became partly paid for use and at some parts the curation is arbitrary	http://www.genome.jp/kegg/
Ingenuity Pathway Analysis^97^	A complete user-friendly pathway analysis tool, which even capable to predict the final outcome	Capable of sophisticated analysis, commercial	https://www.qiagenbioinformatics.com/products/ingenuity-pathway-analysis/
Molecular Signature Database^98^	The Broad Institute's pathway signature collection	Different molecular signatures can be determined according to user, easy compatibility with GSEA	http://software.broadinstitute.org/gsea/msigdb

All pathway-based enrichment methods have a curation bias: the most important genes or pathways are well researched so they tend to have more ontological entries, or in the case of pathways, more member genes. Enrichment analysis by default does not give entirely novel mechanisms of action because some understanding about the genes involved needs to be provided to annotate them with meaningful pathways. However, the method contextualises experimental findings with the currently available biological insight. It is a common problem to receive a large number of ‘enriched’ pathways from such analysis, so the choice of background correction and filtering for relevant mechanisms is frequently employed. Kim *et al.* used the GOrilla^103^ tool to examine altered pathways after MPP+ induction in human neuroblastoma cells, finding different nucleosome assembly Gene Ontology biological processes to be enriched in a time-dependent manner.^74^

After enriched pathways have been found, a pathway map can be formed to shed light on causative biological events. An example is the work of Bell *et al.*,^104^ where the authors conducted a DEG analysis by determining FC from TG-GATEs.^18^ These DEGs were used to calculate the enriched pathways in Reactome constructing a “computationally predicted adverse outcome pathway” for each compound for a specific pathological phenotype. The usefulness of the method was validated with the example of the fatty liver disease caused by carbon tetrachloride.

**Fig. 3:**
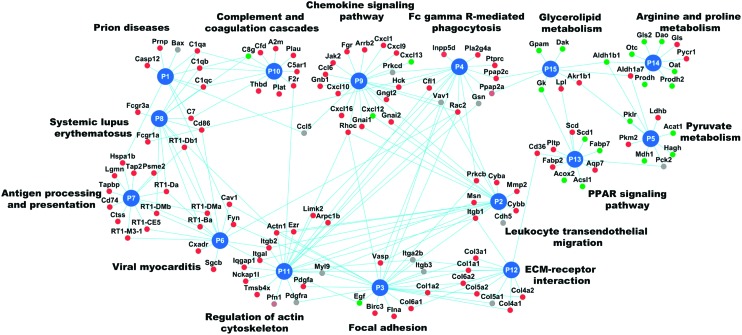
An example network of enriched KEGG pathways of compound-induced differentially expressed genes relating to liver fibrosis. Of the 15 pathways shown, down-regulated pathways were predominantly metabolism related; whereas up-regulated pathways were related to processes associated with liver fibrosis, for example, the focal adhesion pathway is pathway 3 (P3) and also immune related pathways are depicted (P7-antigen processing or P9-chemokine signaling). The authors hypothesised that the metabolic pathways could be related to external factors (*e.g.* altered food intake) or an indication of reduced liver function. Genes with an average fold change >1.5 are in red, <0.75 are in green, and the remaining in grey. P*X* represents pathway number *X* and is represented in a blue circle. DOI: ; 10.1371/journal.pone.0112193.g003.

Abdul Hameed *et al.* used protein interaction networks (see relevant section below) and pathway analysis to find toxic pathways involved in liver injuries in rats *in vivo*.^101^ They showed decreased metabolism in the liver but increased inflammatory pathway activity and increased expression of genes in fibrosis-relevant pathways (Fig. 3 – replica from ref. 101).

Pathway analysis forms the basis of most toxicogenomics analyses. The results of it may not be trivial to understand; determining mode-of-action from hundreds of DEGs and hundreds of enriched pathways is not always possible. As such, further methods have been developed to annotate experimental gene expression data with additional context.

### Compound signature matching methods

A further development in toxicogenomic methods is signature-matching approaches, where compound-induced gene expression signatures are evaluated against a pre-existing compound signature library in order to make predictions about their potential toxicity. Compound signature matching methods have been used in a broad range of applications from side effect prediction^19^ to drug repurposing,^105^ based on the assumption that compounds inducing similar gene expression signatures will have similar effects in a biological system. In the field of toxicity, this allows the matching of test compounds to those with known toxicity profiles, or that have a known mechanism of toxicity. Importantly, basing the comparison on transcriptomic read-outs, rather than compound structure, may lead to a similarity profile very distinct from that obtained by structural similarity.^87^

Transcriptomic profiles of compounds can be obtained from compound signature collections such as CMap^19^ or LINCS (Table 1).^51^,^52^ As these collections measure compound-induced gene expression *in vitro*, a greater number of compounds can be queried when compared to the *in vivo* measurements in the toxicity-specific databases mentioned above such as TG-GATEs or DrugMatrix. In this part of the review, we therefore will focus on the *in vitro* databases CMap and LINCS, and their utilization in understanding and predicting compound toxicity.

**Fig. 4:**
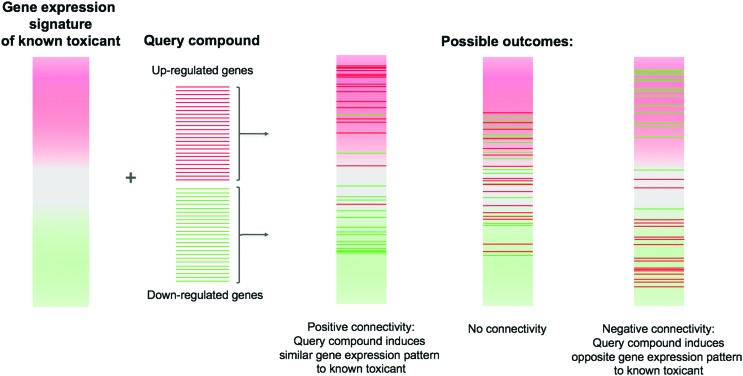
Connectivity mapping for compound signatures. Lists of up- and down-regulated genes resulting from perturbation by a compound are compared against gene expression signatures from reference compounds. Positive connectivity (where the genes up-regulated by the compound under test are also up-regulated by the reference compound, and similarly for down-regulated genes) indicates that the two compounds induce similar gene expression profiles; negative connectivity indicates the opposite.

Using these signature libraries, researchers can measure the similarity between compounds in gene expression space. A widely-used method to do this is connectivity mapping,^19^ which takes into account that the most strongly differentially expressed genes are likely to be more informative than the entire transcriptome. Connectivity mapping describes the enrichment of a ‘query’ signature (for instance, a list of the top most up- and down-regulated genes) against a reference transcriptomic profile (*e.g.* of a known toxicant) (Fig. 4). This is measured by a connectivity score based on the Kolmogorov–Smirnov statistic for the up- and down-regulated genes of a query compound. The original paper describing CMap illustrated how connectivity mapping could be used to elucidate the mechanism of action of a compound or predict side effects such as weight gain,^19^ and several early applications of connectivity mapping in toxicology are covered in a mini-review by Smalley *et al.*^106^

More recently, a case study of the use of gene expression data in drug discovery projects described how such an approach was used to evaluate the toxicity of compounds inhibiting PDE10A, an antipsychotic target.^107^ Expression profiling was carried out on human embryonic kidney (HEK293) cells for the compounds under development, revealing a strong downregulation of tubulin genes. The level of tubulin downregulation correlated with high levels of micronuclei formation, suggesting that the tubulin genes could be used as a predictive signature of micronuclei formation. These signature genes were then used to query the Connectivity Map to find compounds with similar patterns of gene expression. Four of the five most similar compounds returned by this approach were known genotoxic compounds, one of which is commonly used as a positive reference in the micronuclei formation test. This result was used to suggest subsequent transcriptomic experiments, which validated the link between the tubulin genes and micronuclei formation. The authors suggest that transcriptomic profiling could therefore provide an early indicator of potential genotoxicity, allowing compounds to be excluded well before the micronucleus test, which is usually performed late in the drug development pipeline.^107^

As well as testing for connectivity to known toxic compounds, compound signature similarity can be used to infer mechanisms of toxicity.^108^ One case study involves the use of connectivity to predict novel hERG (human ether-a-go-go-related gene) K+ channel inhibitors.^88^ Inhibition of the hERG channel leads to an increased risk of sudden cardiac death,^109^ but known hERG inhibitors are diverse with respect to their structure and primary targets, causing difficulty in the computational identification of potential inhibitory compounds.^88^ In order to investigate whether transcriptomic signatures could provide a signal of hERG inhibition, CMap profiles of 673 drugs including 119 known hERG inhibitors were clustered using affinity propagation, a clustering algorithm based on the idea of communication between data points.^110^ Similarities in the profiles of structurally diverse known hERG inhibitors were used to create a transcriptomic profile of hERG inhibition in different cell lines, revealing differential expression in groups of genes enriched for diverse processes including cholesterol and isoprenoid biosynthesis and the cell cycle. Clusters enriched for hERG inhibitors predicted novel inhibitors that showed significantly greater inhibition than randomly selected compounds, illustrating how CMap data can be used to generate signatures of toxicity based entirely on public data.

As well as the general issues faced in the analysis of gene expression data (as described above), there are further considerations arising from the use of *in vitro* cell line measurements in the largest compound-induced signature databases, CMap and LINCS. It is known that gene expression in cell lines does not always correlate closely with that measured in the corresponding organ;^100^ further, the gene expression response to compounds may be affected by the type of cell line used.^108^ Differences in cell line response, as well as between dosage and time point of compound administration, must therefore be taken into account when analysing this type of compound-induced signature. Nonetheless, as demonstrated in this section, signature-matching approaches can be a powerful tool for early hypothesis generation before later *in vivo* validation.

### Utilizing biological networks for toxicogenomic studies

**Fig. 5:**
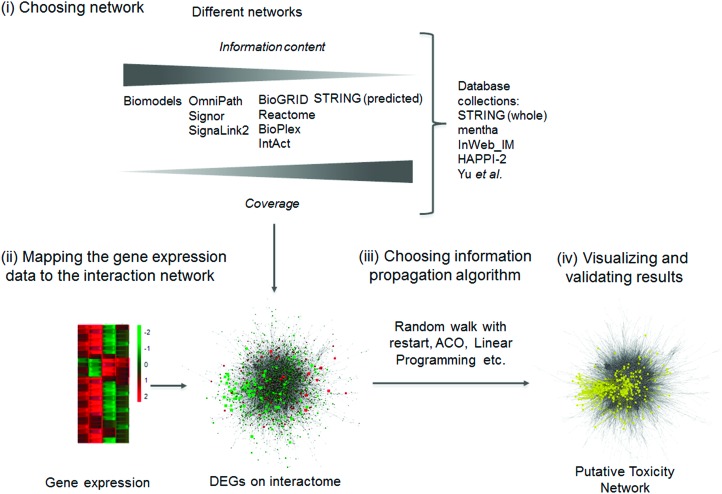
Inferring mode of toxicity using network biology. The initial step (i) is to select a network with appropriate coverage and information content according to the question at hand. Subsequently, (ii) gene expression data needs to be merged with the selected PPI network database. Following this, (iii) an algorithm connects the differentially expressed genes in the network. The resulting putative toxicity networks can be depicted and suggest the mode of toxicity for a compound (iv in yellow), but further experimental validation is required to confirm the prediction. DEG – differentially expressed gene, ACO – ant colony optimization. For the different databases, see Table 4.

Biological interaction networks, such as protein–protein interaction or signaling networks, can be useful tools to decipher the mechanism of toxicity. Biological networks can be directed when we know which way the information flows from one node (protein, miRNA, small molecule, gene, *etc.*) to the other; or undirected when this information is unknown or it has no meaning, *e.g.* proteins forming a complex. Biological networks, especially directed signaling networks, allow us to follow the cellular response of a compound treatment from the compound's target to the differentially expressed genes. Different biological networks and databases are compiled from various data sources with varying coverage and information content, *e.g.* whether a network is directed or whether an interaction is inhibitory or excitatory (signed) (Fig. 5(i) and Table 4). The most commonly used biological networks are protein–protein interaction (PPI) networks, whose nodes represent proteins and edges represent interactions *i.e.* the binding of one protein to another. The researcher in every toxicogenomics project has to determine whether they want to look into a specific toxicological process deeply or map a general response and choose the network accordingly.

**Table 4:** Network resources for toxicogenomic studies

Network resource name	Description	Number of interactions in human	Species	Web address
Biomodels^111^	Small-scale dataset containing rate-related interactions. Varying coverage by model type.	Varies in scale 10–1000	Various	https://www.ebi.ac.uk/biomodels-main
NRF2Ome^114^	Small scale manually curated oxidative stress and NRF2 response specific database. Interactions are directed and signed.	289 NRF2 specific PPI	Human	http://nrf2.elte.hu
OmniPath^89^	Manually curated partly directed and signed signaling database which integrates other high quality interaction sources.	50 247	Human	http://omnipathdb.or/
SignaLink2^115^	Multilayer signaling database with regulations and predicted interactions. The manually curated interactions are directed and signed.	1640 high confidence PPI	Human, fruit fly, *C. elegans*	signalink.org
Signor^116^	Manually curated pathway interactions with directions and signs.	19 312	Human, mouse, rat	signor.uniroma2.it
Reactome^117^	Manually curated large scale reaction centered pathway database, which focuses on protein complexes, but the interactions are directed and signed.	11 426	Different organisms including, human, rat, mouse	www.reactome.org
HPRD^118^	Historic, no longer updated database of manually curated undirected interactions.	41 327	Human	www.hprd.org
Bioplex^119^	Large-scale immunopurification and mass spectrometry based protein interaction database.	70 000	Human	http://bioplex.hms.harvard.edu
BioGRID^120^,^121^	Genetic and protein interactions from low and high throughput publications.	406 487	Multiple species including human, rat, mouse	https://thebiogrid.org
IntAct^120^,^121^	Large scale protein interaction database collection.	310 183	Mostly human, but contains other species data as well including mouse	https://www.ebi.ac.uk/intact
InWeb_IM^122^	Large scale collection of PPI datasets with orthological predictions.	625 640	Human	https://www.intomics.com/inbio/map/#home
HAPPI-2^123^	Large database collection of protein interactions with a confidence score.	2 922 202	Human	http://discovery.informatics.uab.edu/HAPPI
mentha^124^	Scored collection of interactions from publications and databases.	309 088	Multiple model organisms including mouse, rat, and humans	http://mentha.uniroma2.it
STRING^125^	Large scale predicted and curated interactions database. Uses text mining and orthology to cover the interactions in different species.	11 353 056	Multiple different organisms	https://string-db.org
Yu *et al.*^126^	Inferred high-confidence human protein–protein interactions from multiple data sources.	80 980	Human interactions	https://bmcbioinformatics.biomedcentral.com/articles/10.1186/1471-2105-13-79

The interactions from biological networks can be characterized based on the source of the interaction and the types of annotation available, such as the direction, strength, kinetics, and sign (inhibitory or activating) of an interaction. While the ultimate aim for biological network studies is to model the whole cell and organism using detailed quantitative interactions, as of yet such detailed models are only available for a few genes or proteins in the BioModels database,^111^–^113^ rendering them unsuitable for toxicogenomics modelling at the current stage.

Manually curated databases typically contain somewhat less detailed information. Such databases include HPRD^118^ for undirected human interactions, and OmniPath^89^ or Signor^116^ for directed and signed signaling information. Reactome^117^ assembles pathways from curated interactions, but in some cases the directionality is impossible to define. Such manually curated databases are biased toward well-studied proteins and interactions, but these tend to be more accurate than high throughput databases. On the other hand, some databases contain information obtained from large, high-throughput experiments, such as BioGRID,^120^ BIOPLEX,^119^ and MINT.^121^ Although these databases contain many interactions, not every interaction is manually checked, so the confidence is usually lower. Most of the experiments are derived from yeast two hybrid model systems, which do not cover nuclear interactions or interactions in the cell membrane; an exception is BIOPLEX, which uses immunoaffinity purification with mass-spectrometry which gives unbiased, reliable data, but cannot differentiate the exact formation of complexes. However, the advantage of such high throughput databases is that they provide unbiased and large-scale information.

Other interaction databases aim to aggregate information from multiple sources, such as STRING, InWeb_IM and HAPPI.^122^,^125^,^127^ Appropriate filtering of such databases can make them applicable to answer toxicological questions, but it should be noted that merging different databases can increase the noise as well as the coverage. Consistency of data and annotations from multiple sources is a frequently recurring problem in this case.

To utilise biological networks (which are chosen according to relevant criteria, as in Fig. 5 step i), DEGs or transcriptomic signatures are first matched to proteins (Fig. 5 step ii). Identifier matching tools, like the UniProt retrieve^128^ service or the Protein Identifier Cross-Reference resource,^129^ can help to do this step.

The next step is to identify which functions these proteins affect in the network (Fig. 5 step iii). Most methods use random walk with restart algorithms, including ENRICHNET,^130^ NETPEA,^131^ and NetWalk.^132^ A related approach is the heat diffusion based algorithms such as HotNet2^133^ or DMFIND.^134^ Random walk with restart begins from the protein equivalents of the selected DEGs and walks around the PPI graph, with a random chance of restarting, to see which proteins can be reached from the start. In a case study testing the NetWalk algorithm, Komurov *et al.* used a unified PPI and transcription factor-target gene network.^132^ They captured the cell cycle arresting function of p53 to sublethal doses of doxorubicin and the apoptosis induction of p53 at lethal doses in MCF7 cell lines. HotNet2 was developed and successfully used for module assignment in pan-cancer data. It detected 16 such modules including the p53 and the NOTCH signaling module in multiple cancers.^133^

A more sophisticated method to find the affected proteins in the network is the Ant Colony Optimization (ACO),^135^ where the random walker (ant) leaves a ‘pheromone trail’ behind it, which increases the probability that the next ant will walk the same path. The strength of the pheromone trail depends on a function of the visited nodes in the graph. For example, if an ant reaches another signature node – such as a DEG – then the next ant can walk the same path and connect the new signature nodes with a path. It is an extension of random walk methods because ACO can connect, in the network sense, distant paths and not just discover the neighbourhood of the signature nodes.

In the toxicogenomics field, ACO was successfully used by Abdul Hameed *et al.*^101^ to uncover how toxicants can cause liver fibrosis through extracellular matrix bound growth factors. The authors determined differentially expressed genes using the rank product method from DrugMatrix^17^ data and also clustered them based on their co-expression in liver fibrosis. The differentially expressed genes and the co-expressed genes from the enriched clusters were mapped to a previously inferred and rescored high quality PPI network.^126^ KeyPathwayMiner,^136^ an ACO implementation for network analysis, was next used to construct the liver fibrosis-associated network. The network was then clustered with the EAGLE^137^ algorithm implemented in the Clusterviz Cytoscape plugin^138^ to find the network module most highly correlated with liver fibrosis. This method was shown to uncover novel interactions in liver fibrosis, which could not be revealed using pathway enrichment of co-expressed and differentially expressed genes. In this module, the extracellular matrix compartments and bounded growth factors were overrepresented, which was validated *via* independent data sets. The utilization of a PPI network enhanced the scope of the analysis, because it incorporated the indirectly affected genes, whose expression themselves was unchanged.

With network biology tools, the feedback effects can be followed from the targets of toxicants to the measured gene expression signature through transcription factors and a putative adverse outcome pathway can be constructed. Melas *et al.*^102^ achieved that in the case of drug-induced lung injury. They used Reactome as a source of protein interactions and a collection of transcription factor target data to connect gene expression signatures of drugs from CMAP with transcription factors. This analysis used a modified Integer Linear Programming algorithm.^139^ Integer Linear Programming is a tree-growing algorithm that finds the shortest tree between two sets of nodes in a directed graph. In this study, the algorithm was modified by adding transcription factors as a third set of nodes that have to be reached. The trees start to grow from the targets of toxicants, through transcription factors, to the differentially expressed genes. These trees formed the putative adverse outcome pathways for specific compounds. They validated their method with an independent pathway growing algorithm and random controls. The developed trees identified central apoptosis relevant proteins such as p53, CASP3, BCL2, BAX, CASP6, CASP8, CASP9 *etc.* and key signaling proteins such as FOS and JUN. Furthermore, these paths showed potential targets to avoid drug-induced lung injury and the authors tested specified drugs, which counteracted the lung injury as a toxic endpoint.

Biological networks can help to uncover hidden modes of toxicity with the help of gene expression data. They work with the assumption that the level of a transcript's expression is highly correlated with the amount of protein. However, this assumption is not absolutely true in all cases.^140^ To choose a proper biological network for a toxicogenomics study, the researcher must choose between information content and coverage. Nonspecific interaction databases with large coverage are suitable to generate unbiased hypotheses in toxicogenomics. If the coverage is not so important but the information content and reliability is a key issue, then manually curated database such as Signor or OmniPath or even smaller databases like NRF2Ome^114^ may be more appropriate. If kinetic modelling is the aim then the researcher must initially look up a relevant model from the BioModels database. The middle ground could be a database such as Reactome to show specific toxic responses with an appropriate coverage of signaling in humans and model organisms.

### Co-expression network methods

Co-expression network analyses are methods that utilise the entire measured transcriptome to help determine gene function and mode of action. They are divided into two main categories, namely data-driven and knowledge-based methods. In both cases, co-expression analyses rely on the hypothesis that highly correlated genes are biologically related.

An early co-expression method was used by Deng *et al.*,^141^ where the coexpression network was determined by a method called Context Likelihood of Relatedness.^142^ This method uses mutual information (MI) to create a similarity network of genes by estimating the MI between two genes against a background distribution, taken to be the distribution of MI scores per gene.

Using this method, the authors found that human and rat hepatocytes respond with a similar gene network when exposed to 2,4,6-trinitrotoluene (TNT). The similarity of this response is crucial, as animal models are required to be representative of the human response to be useful to anticipate compound toxicity in man.

**Fig. 6:**
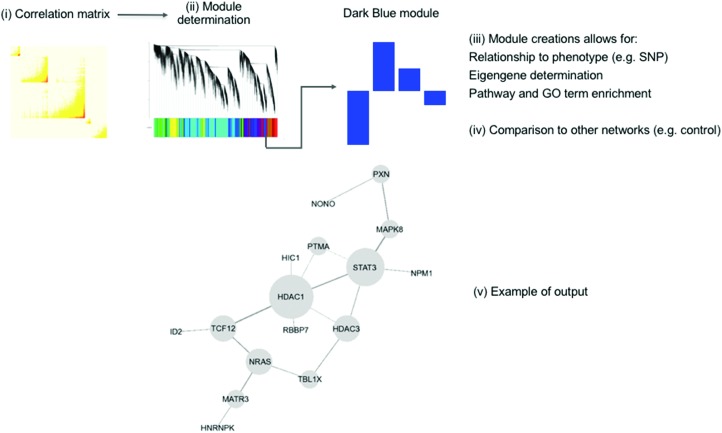
An overview of the WGCNA method showing the four main steps. First, the correlation between gene expression values is calculated as a matrix (i). This is then used to determine modules (ii), which can be related to external information (iii), such as a phenotype, as well as being compared to other coexpression networks (iv). The modules found to be associated with this external information can form hypotheses about its generation. (v) Shows an example of WGCNA method using MPTP toxicity in mice. The HDAC1 subnetwork is from the FANTOM4 regulatory network.^147^ The genes shown were all connected to HDAC1 in the co-expression network. The authors state that the connections between modules have been preserved through the reduction of dimensionality. This figure is used from Maertens *et al.*;^147^ DOI: ; 10.1007/s00204-015-1509-6.

Another popular method is ‘Weighted Gene Co-expression Network Analysis’ (WGCNA) which was first published in 2005 and later released as an R package.^143^,^144^ This method can be split into four major steps, which are visualised in Fig. 6: (i) the generation of the co-expression network, (ii) the definition of co-expressed gene modules, (iii) the relation to external information (*e.g.* clinical data, other-omics data, GO terms and pathways), and (iv) the determination of conserved/changed elements between different networks. The first step, setting up the network, is computing the correlation between each probe-set/gene and raising the resulting matrix to a soft power. This soft power is used to reduce noise and optimise the scale-free property of the network. Next, modules are created by creating a dissimilarity matrix from the topological overlap matrix and these are then identified by hierarchical^145^ or k-means clustering.^146^

There have been several uses of this method. Guo *et al.* analysed microarray data from mice exposed to chloroprene at both carcinogenic and noncarcinogenic doses.^148^ Seven hub genes (*i.e.*, an interpretable number) were determined to be vital for carcinogenesis, providing potential biomarkers and drug targets. The WGCNA method was also used, in addition to other methods, in the study of liver fibrosis.^101^ Based on the DrugMatrix database, this analysis defined toxicity using a cutoff of 1 in the ‘liver periportal fibrosis’ histopathology score. Known and new genes were found to be associated with liver fibrosis, which helped to shed light on the relevant mode of toxicity. Genes such as TIMP1, APOA1, CTGF, LGALS3, TGFB1, and MMP-2 are in the same module and are annotated with ‘liver cirrhosis’ in the CTD (liver fibrosis is not a curated term) and ‘Extracellular matrix (ECM) organisation’ and ‘wound healing’ GO terms. Genes not previously associated with liver fibrosis include LGMN, which is a cysteine protease that functions in ECM remodelling, and PLIN3, which is known to play a role in the pathogenesis of steatosis and PGE2 production. This study also linked two known toxicants, carbon tetrachloride, and lipopolysaccharide, to liver fibrosis even before the histopathological lesion became visible. This demonstrates that WGCNA, in conjunction to other methods, can reveal early-stage biomarkers for toxicity in the form of up- and downregulated genes.

This method was used to delve into the pathway of toxicity of MPTP in mice.^147^ Five modules were found to be significant. These were integrated with the FANTOM4 gene regulatory database to generate a network, as shown in Fig. 6 part v.^149^ This analysis confirmed the known mechanisms of toxicity of MPTP as well as suggesting the SP1 transcription factor as a critical player in MPTP response. This has wider implications for the study of Parkinson's disease, for which MPTP toxicity is used as a model.^150^

Direct association between phenotype and compound induced gene expression using WGCNA was performed by Sutherland *et al.*^151^ Using both DrugMatrix and TG-GATEs, modules were determined and enriched with GO terms and histopathological scores. Several case studies were performed, including one that identified a novel mechanism of hepatotoxicity involving endoplasmic reticulum stress and Nrf2 activation. Additionally, it was shown that using co-expression network analysis increased the number of phenotype-gene associations, both novel and established.

A second method for analysing co-expression networks is the iterative signature algorithm (ISA). This method is reliant on starter seeds, which are typically gene sets from hierarchical clustering although they may also be randomly generated.^152^ Modules are refined iteratively by adding/removing genes at each step; gene and condition threshold parameters determine the size and stringency of the modules created. In contrast to WGCNA, overlap of genes and samples between modules is permitted in this method.

In one recent comparative study, Tawa *et al.* used multiple algorithms to find signatures associated with ‘chemically induced liver injuries’.^153^ Using the DrugMatrix database, the authors also combined clinical pathology, organ weight changes and histopathology to define 25 diverse toxic endpoints. Modules were created with a variety of different approaches, namely hierarchical clustering, support vector machines and PPI networks, using the most highly differentially expressed genes associated with a particular liver injury, and compared to the results obtained from ISA. The ISA method outperformed other methods in that it (re-)created modules that showed enrichment of liver injury from gene–disease relationships and biomarkers provided by the comparative toxicogenomics database (CTD).^48^ These genes include Sod2, Gulo and Car3 (associated with periportal lipid accumulation), and Obp3 and Rgn (associated with periportal fibrosis). This analysis was validated using the Open TG-GATEs database.^18^

ISA has also been used to predict acute kidney injury (AKI).^154^ In this case, the modules created were specific for the cause of kidney injury, as they were activated by specific compounds and contained ‘acute kidney injury’ relevant genes. These modules were used to create a biomarker list comprising 30 genes for acute kidney injury potential which could be used before the injury actually occurs. These biomarkers were validated by comparison with modules comprised of random genes as well as additional gene expression data from GEO. The genes previously associated with AKI were found using this method, including Havcr1, Clu, and Tff3. Novel genes suggested to be involved in AKI were those that co-expressed with Havcr1, including Cd44, Plk2, Mdm2, Hnmt, Macrod1, and Gtpbp4. These were also found to be co-expressed in a non-chemically induced kidney injury model, which implies a nonspecific response to injury.

While co-expression methods clearly have significant potential in analysing and predicting compound toxicity, they are reliant on the assumption that highly correlated genes are biologically related. Correlation does not mean causation and this must be considered when determining modes of toxicity. Another issue to be considered is that the methods are dependent on determining correlations between genes, and so a suitable minimum of replicates is required: the WGCNA method designer suggests a minimum of 15 (sample and control). However, as shown in the above evidence, it appears that such methods represent a sensible and state-of-the-art way to reduce large amounts of data down to informative gene sets.

## Conclusion & future perspectives

**Fig. 7:**
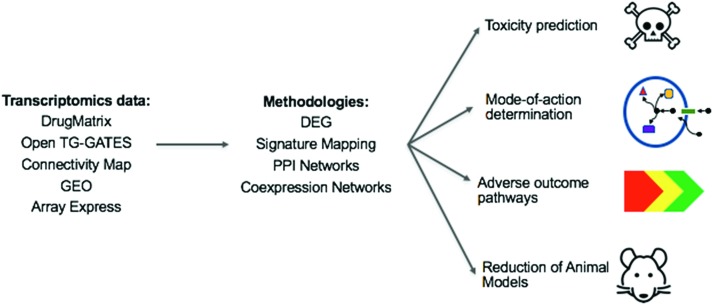
The overview of the current and potential impact of toxicogenomics research. From the listed toxicogenomic databases, provided sufficient data is available, the toxicity of a compound, its mechanism of toxicity, and a related Adverse Outcome Pathway can potentially be inferred using the methods reviewed here. With increasing data available, and increasing sophistication of methods, the aim is that this will, over time, result in decreased animal testing and decreased amount of failures during drug development. (TG-GATEs: Toxicogenomics Project-Genomics Assisted Toxicity Evaluation System, GEO: Gene Expression Omnibus, DEG: Differentially Expressed Genes, PPI: protein–protein interactions).

With this review, we took a snapshot of the state-of-the-art methods in the evolving field of toxicogenomics. Toxicogenomics can be used to address two of the most important issues in toxicology: elucidation of a compound's mode of toxicity, *i.e.* understanding why it is toxic, and prediction of whether a compound is toxic or not. This can affect many areas, as summarized in Fig. 7.

Major limitations of the toxicogenomics field are the available data sources, with respect to the chemical space (compound coverage) and the availability of gene expression data (tissue/cell line, dose, time point *etc.*), as well as the availability of toxic endpoint annotations.

Often data available are not entirely the ‘right’ data for the intended purpose: this is exemplified by the use of cell lines to understand compound-induced gene expression in databases such as CMap, where cell lines do not fully capture the response of a whole organism to a compound. Currently, the best model organisms, which provide high-level phenotypic readouts, are mice and rats. However they do not have exactly the same physiological parameters as humans,^155^*e.g.* their immune system reacts to compounds differently.^156^

A big issue in any toxicological study is that organisms respond to a wide range of perturbations with a similar response: stress.^157^ Different stress responses are visible in the gene expression response of cells to compound treatments, but it is still often hard to distinguish a compound-specific signal.^158^ Coexpression network methods, amongst other toxicogenomics methods, can elucidate the similarities and the differences of each response for each specific compound, and so help to identify the generic stress response.^151^,^152^ As the field progresses, the generic stress response will be teased apart using specific mode of action studies to provide clarity on toxic events.

Despite the limitations of the field currently, toxicogenomics methods are already seeing wider recognition and adoption by the pharmaceutical industry, such as in deriving Adverse Outcome Pathways.^159^ They can help determine the molecular initiating events and can reveal the cascade of events leading to the phenotypic manifestation of toxicity.^88^,^102^,^107^

Early-stage gene expression markers for toxicity found using toxicogenomics methods, will be crucial in deciding which compounds to pursue during drug development.^107^ This could help to reduce animal testing^5^ by stopping *in vivo* experimentation with compounds that are unacceptably toxic.

We think in the future we will see the reviewed methods extending to transcriptomic data drawn from organoids^160^ and microfluidic bound organs on chips.^161^ These technologies will be able to model the human body with more reliable absorption and distribution rates compared to animal models or cell lines.^162^ An orthogonal extension of toxicogenomics methods will be their application to *in silico* human models, the foundations of which have already been laid by the biomodels^111^,^163^ highlighted in this review.

In conclusion, toxicogenomics can help to understand both the mechanism of toxicity and predict compound toxicity. As the field progresses, it will help to reduce animal testing, reduce late-stage drug development failures due to toxicity and have a direct impact on decisions in the clinic.

## Conflicts of interest

There are no conflicts of interest to declare.
